# Cottage Hospitals and Medical Fees

**Published:** 1896-12-12

**Authors:** 


### COTTAGE HOSPITALS AND MEDICAL FEES.

We have received a long correspondence between Mrs. Drax, of Charborough Park, Wareham, and Messrs. Parkinson and Fawcett, surgeons, Wimborne, in regard to a charge made by the latter for attendance upon a servant of Mrs. Drax while a patient in the Wimborne Cottage Hospital. The rules of the cottage hospital say (Rule 13), "The hospitals shall be for the use of the working classes of the town and neighbourhood, who shall pay not less than 2s. 6d. per week (or upwards, at the discretion of the visitors), but persons in better circumstances may be admitted by the visitors at special charges and by special arrangement, and in the case of such persons any question of remuneration to the medical officers they (the medical officers) are authorised to settle with them." It seems to us quite clear that the patient in question was not "admitted by the visitors at special charges and by special arrangements," and therefore does not come under the category of those in regard to whom the medical officers may charge fees; at the same time we cordially agree with the secretary, General Maclean, that one cannot consider that "a servant in receipt of good wages, board, and lodging, with a claim, if discharged, for a month's wages and board, can be put on the same footing as a man (or woman) on weekly wages and having a house to pay for, possibly a family dependent on him." It is clear that the patient in question was improperly admitted unless she was admitted as an emergency, in which case, however, according to Rule 11, the admission was only valid till the following visitors' meeting, when a proper understanding should have been arrived at with her employer. If the rule as to admission had been adhered to the medical officer would have had a perfect right to make a charge.

